# Interferon γ-induced GTPase promotes invasion of *Listeria monocytogenes* into trophoblast giant cells

**DOI:** 10.1038/srep08195

**Published:** 2015-02-03

**Authors:** Masato Tachibana, Masanori Hashino, Kenta Watanabe, Takashi Shimizu, Masahisa Watarai

**Affiliations:** 1 The United Graduate School of Veterinary Science, Yamaguchi University, Yamaguchi, Japan

## Abstract

*Listeria monocytogenes* is well known for having the ability to cross the placental barrier, leading to fetal infections and abortion. However, the mechanisms leading to infectious abortion are poorly understood. In this study, we demonstrate that interferon γ-induced GTPase (IGTP) contributes to the invasion of *L. monocytogenes* into trophoblast giant (TG) cells, which are placental immune cells. Knockdown of IGTP in TG cells decreased the relative efficiencies of *L. monocytogenes* invasion. Moreover, IGTP accumulated around infected *L. monocytogenes* in TG cells. Treatment of TG cells with phosphatidylinositol 3-kinase (PI3K)/Akt inhibitors also reduced bacterial invasion. PI3K/Akt inhibitor or IGTP knockdown reduced the amount of phosphorylated Akt. Monosialotetrahexosylganglioside (GM1) gangliosides, lipid raft markers, accumulated in the membrane of *L. monocytogenes*-containing vacuoles in TG cells. Furthermore, treatment with a lipid raft inhibitor reduced bacterial invasion. These results suggest that IGTP-induced activation of the PI3K/Akt signaling pathway promotes bacterial invasion into TG cells.

L*isteria monocytogenes* has the ability to cross the intestinal, blood-brain, and placental barriers, leading to gastroenteritis, meningoencephalitis, and maternofetal infections, respectively. Maternofetal infection results in abortion occasionally. A key feature of the virulence of *L. monocytogenes* is its ability to avoid the killing mechanisms of professional and non- professional phagocytic host cells^1^,^2^. *L. monocytogenes* infections in humans are caused mainly by the ingestion of contaminated food, such as dairy products, raw vegetables, fish, poultry, processed chicken, and beef^3^.

Pregnancy leads to a generalized suppression of the adaptive immune system, typified by significantly decreased cell-mediated immunity and reduced T helper cell (Th) 1 responsiveness^4^,^5^. This immunosuppressed state prevents maternal rejection of the fetus but has the unfortunate consequence of increasing maternal susceptibility to certain infectious agents^6^,^7^. Immunity against *L. monocytogenes* is principally mediated by cellular immune responses because it is an intracellular pathogen^8^. For many other intracellular bacterial and protozoan pathogens, it has been shown that interferon-γ (IFN-γ) is an important component of Th1 immune responses and contributes to control through its ability to stimulate macrophages to kill more microbes. The infectious abortion model using a pregnant mouse is a powerful tool for investigating the mechanisms of bacterial pathogenesis. In our previous study, we demonstrated that abortion-inducing bacteria in human and animals, such as *L. monocytogenes* and *Brucella abortus*, cause abortion in pregnant mice model^9^,^10^. In *Brucella* infection, we found that there was a higher degree of bacterial colonization in the placenta than in other organs, that there were many bacteria in trophoblast giant (TG) cells in the placenta and that abortion was not induced in an intracellular replication-defective mutant^11^. In addition, we demonstrated that infection induced a transient increase in IFN-γ in pregnant mice. This transient IFN-γ production also contributes to infectious abortion, and its neutralization serves to prevent abortion^11^. These studies of *Brucella* infection suggest that bacterial infection of TG cells plays a key role in causing abortion and that TG cells are closely linked to the avoidance of maternal immune rejection.

TG cells are polyploid cells differentiated from trophoblast stem (TS) cells by many morphological and functional developments; they form the fetal component of the placenta^12^. In particular, TG cells play crucial roles in implantation and the formation of a diffuse network of blood sinuses^13^, and promote maternal blood flow to the implantation site in mice^14^. TG cells are essential for the establishment of pregnancy. TG cells in the mouse placenta are parallel to extravillous cytotrophoblast cells in the human placenta^14^. Trophoblast cells also have a phagocytic ability. During implantation, trophoblast cells invade maternal tissue by phagocytosing uterine epithelial cells and stroma^15^. Several molecular mechanisms involved in phagocytosis by trophoblast cells have been reported^16^, however, the complete process remains unclear. It has also been reported that trophoblast cells can phagocytose pathogens and that this activity is enhanced by IFN-γ treatment^17^. Therefore, trophoblast cells may act in a manner similar to that of macrophages in phagocytosis. These studies suggested that trophoblast cells play a role not only in the development and maintenance of placenta but also in the placental defense system.

IFN-γ-induced GTPase (IGTP), also known as Irgm3, belongs to a family of 47 kDa IFN-γ-responsive GTPases (IRG). These family proteins are known to play critical roles in mediating specific resistance to intracellular pathogens including protozoa, bacteria and viruses^18^,^19^,^20^. Because IGTP localizes predominantly to the endoplasmic reticulum, it is assumed to be involved in the processing and trafficking of immunologically relevant proteins^21^,^22^. IGTP has been found to be essential for host resistance to acute infections by the protozoans *Toxoplasma gondii*^23^ and *Leishmania major*^22^, but its antimicrobial mechanism is still not clear.

In the present study, we investigated the role of IGTP in the invasion of *L. monocytogenes* into TG cells. Our results suggested that IGTP induces the activation of phosphatidylinositol 3-kinase (PI3K)/Akt signaling pathway and promotes bacterial invasion into TG cells.

## Results

### IGTP expression is induced by IFN-γ in TG cells

**Figure 1:**
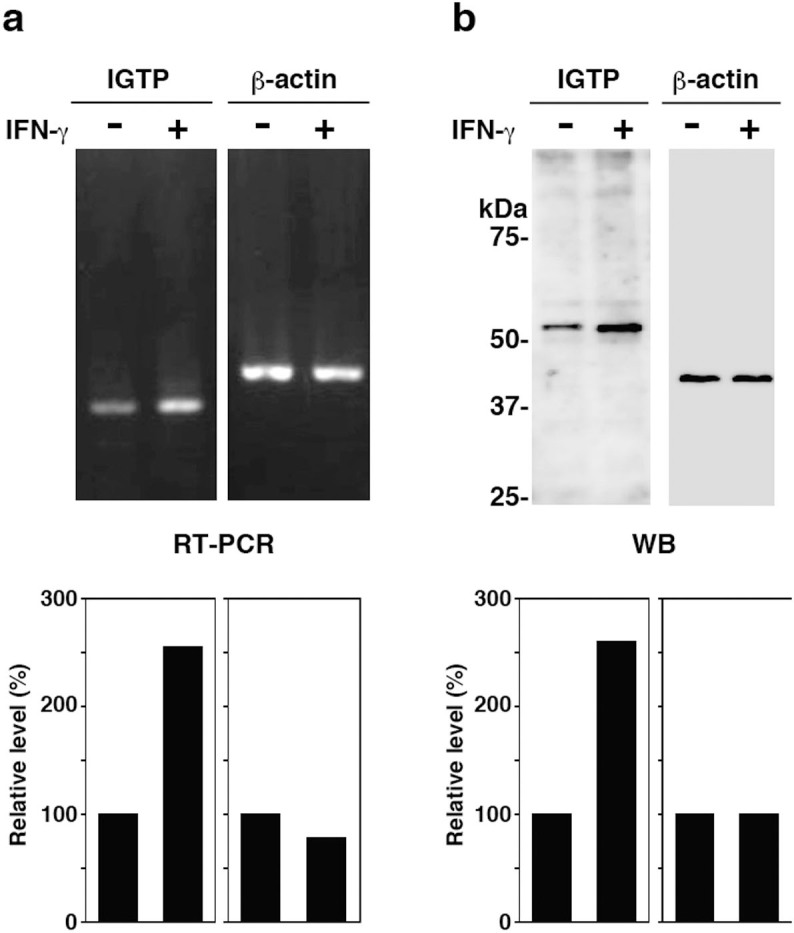
Increased IGTP expression in TG cells treated with IFN-γ. TG cells were treated with or without IFN-γ (3,000 units/ml) for 24 h (a and b); IGTP expression was analyzed by RT-PCR (a) and immunoblotting (b). β-actin was used as a control. The blots were scanned and analyzed using ImageJ software. For each sample, IGTP or β-actin expression observed in the absence of IFN-γ was set at 100% and IGTP or β-actin expression in the presence of IFN-γ was expressed relative to this value (lower panels).

*L. monocytogenes* infects placental cells and induces cell death *in vitro* and *in vivo*^24^,^25^,^26^, which results in infectious abortion occasionally. In a previous study, we demonstrated that IFN-γ promotes bacterial invasion into TG cells^27^,^28^. We speculated that factors induced by IFN-γ should play a key role in the bacterial infection of TG cells. In this study, we focused on IGTP. First, to confirm the expression of IGTP protein and RNA in TG cells, we performed reverse transcription PCR (RT-PCR) and immunoblotting with or without IFN-γ treatment and compared the results. IFN-γ treatment induced IGTP expression at both the RNA (Fig. 1a) and protein (Fig. 1b) level.

### IGTP promotes bacterial invasion into TG cells

**Figure 2:**
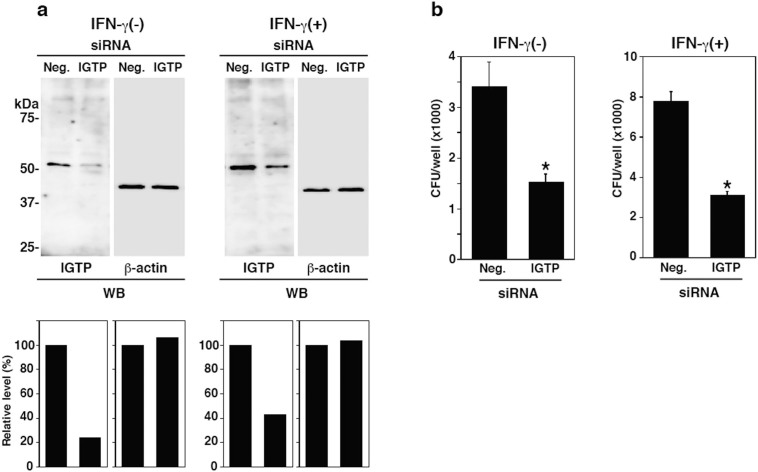
Effect of IGTP depletion on bacterial invasion into TG cells. (a) TG cells were treated for 48 h with siRNA-targeting IGTP, with or without IFN-γ (3,000 units/ml) for 24 h (IGTP) simultaneously during cell differentiation. A negative control sample was treated with AllStars Negative Control siRNA (Neg.). Expression of the indicated proteins was monitored by immunoblotting. β-actin was used as a control. The blots were scanned and analyzed using ImageJ software. For each sample, IGTP or β-actin expression of negative control was set at 100% and IGTP or β-actin expression treated with siRNA-targeting IGTP was expressed relative to this value (lower panels). (b) Bacterial invasion into IGTP-depleted TG cells with or without IFN-γ was studied using a bacterial invasion assay. The efficiency of *L. monocytogenes* invasion into TG cells was increased 2.3 times by IFN-γ treatment. Data are the averages of triplicate samples from three identical experiments and the error bars represent standard deviations. Statistically significant differences compared with the negative control are indicated by asterisks (*, *P* < 0.05).

**Figure 3:**
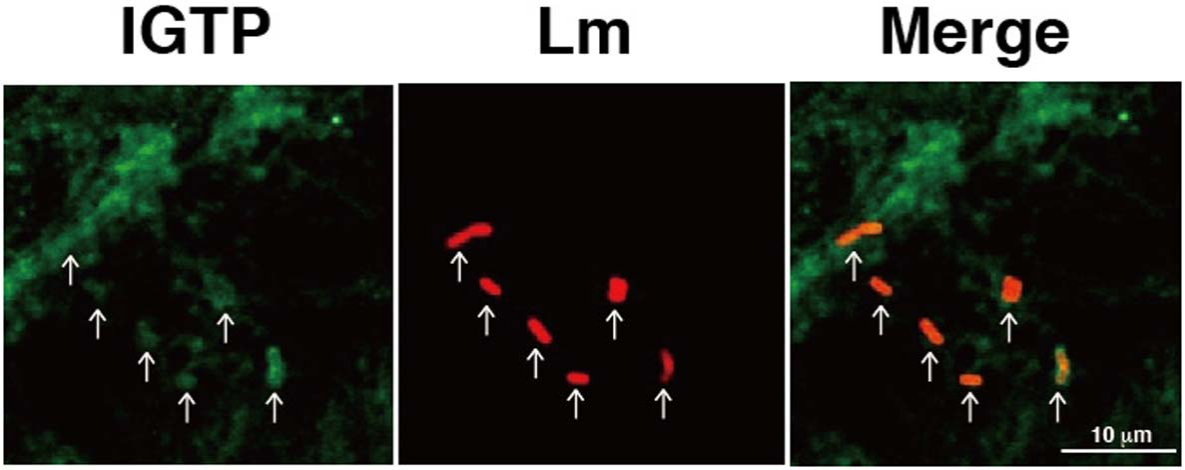
Localization of *L. monocytogenes* and IGTP in TG cells by immunofluorescence microscopy. TG cells were infected with *L. monocytogenes* for 30 min, then fixed and stained for IGTP (green) and intracellular bacteria (red). Arrows indicate bacteria colocalized with IGTP. Scale bar indicates 10 μm.

To examine the effect of IGTP on bacterial invasion into TG cells, we used siRNA to knockdown IGTP in TG cells. Immunoblotting confirmed that IGTP expression levels were reduced in TG cells with or without IFN-γ treatment (Fig. 2a). The relative *L. monocytogenes* invasion efficiencies were significantly reduced by the knockdown of IGTP by siRNA in TG cells, with or without IFN-γ treatment (Fig. 2b). *L. monocytogenes* invasion did not affect IGTP expression (Supplementary Figure S1). Next we examined the localization of IGTP in TG cells 30 min after bacterial infection. IGTP accumulated around infected *L. monocytogenes* in TG cells (Fig. 3). The number of co-localized bacteria with IGTP per 100 intracellular bacteria were counted in 5 times independent experiments and 32.2 ± 10.38% of bacteria co-localized with IGTP. These results suggested that IGTP contributes to bacterial invasion into TG cells.

### IGTP induces the activation of PI3K/Akt in TG cells

**Figure 4:**
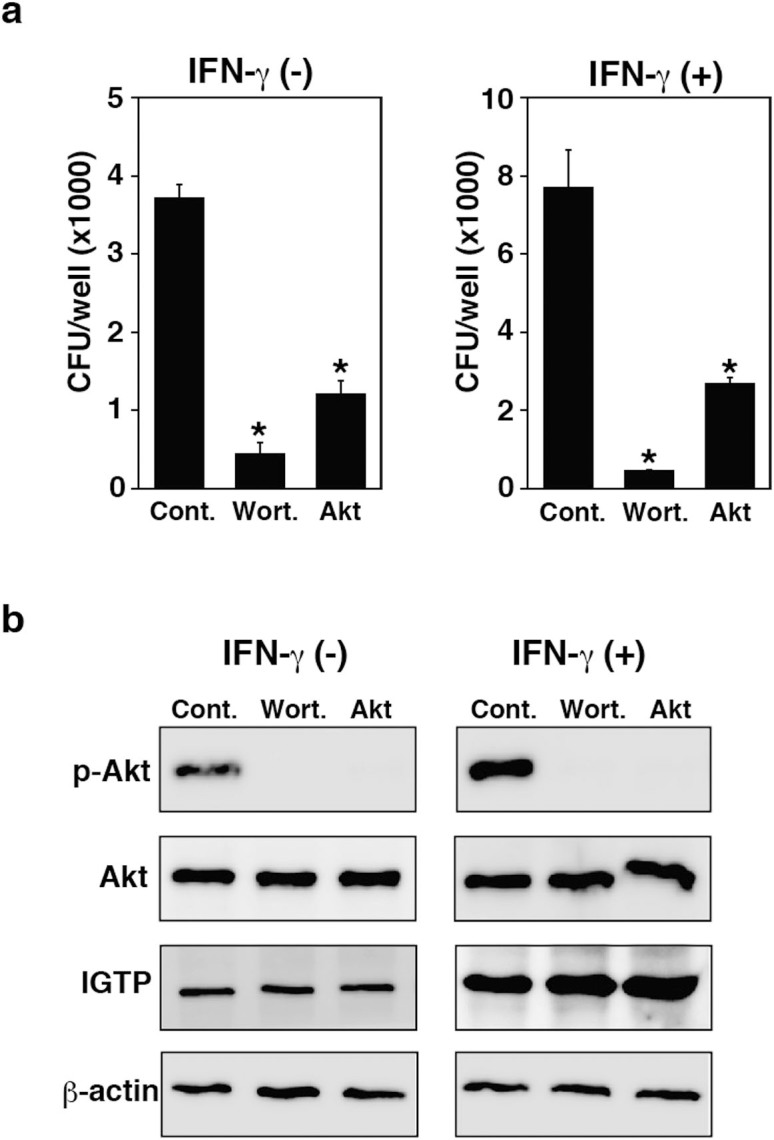
Effect of PI3K/Akt inhibitor on bacterial invasion and phosphorylation of Akt in TG cells. (a) TG cells were treated with wortmannin (Wort.) or Akt 1/2 kinase inhibitor (Akt) for 1 h. Treated TG cells were infected with *L. monocytogenes* in the presence or absence of IFN-γ. Bacterial invasion into TG cells was measured using a bacterial invasion assay. Data represent the averages and standard deviations of triplicate samples from three identical experiments. Statistically significant differences between control and treated groups are indicated by asterisks (*, *P* < 0.05). (b) TG cells were treated for 1 h with wortmannin or Akt 1/2 kinase inhibitor with or without IFN-γ (3,000 units/ml), for 24 h. Expression of the indicated proteins was detected by immunoblotting. β-actin was used as a control. Cropped gels/blots are used in immunoblotting data. Full length images are presented in supplementary information (Supplementary Figure S2).

**Figure 5:**
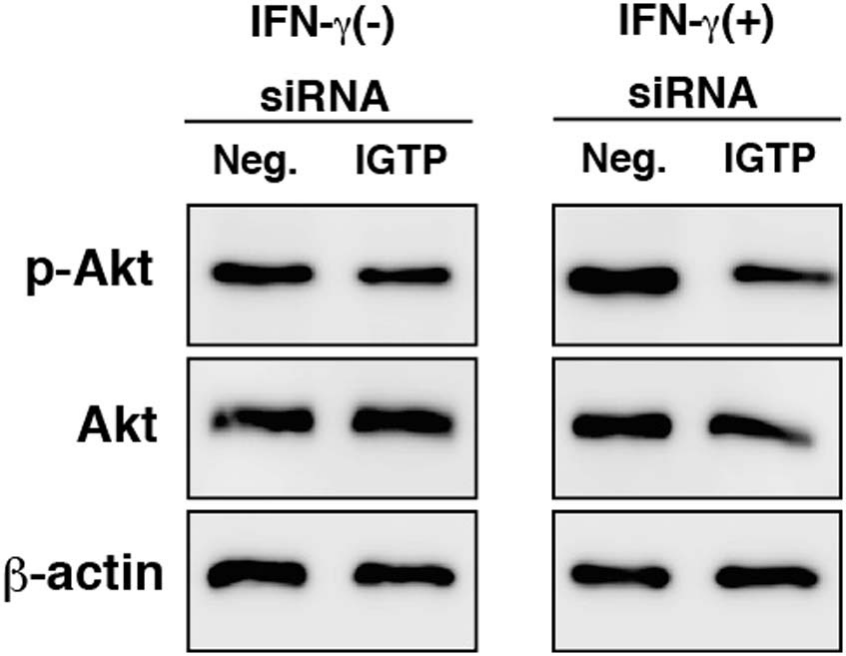
Correlation between IGTP expression and phosphorylation of Akt. TG cells were treated for 48 h with either siRNA-targeting IGTP, with or without IFN-γ (3,000 units/ml), for 24 h (IGTP) simultaneously during cell differentiation. A negative control sample was treated with AllStars Negative Control siRNA (Neg.). Expression of the indicated proteins was detected by immunoblotting. β-actin was used as a control. Cropped gels/blots are used in immunoblotting data. Full length images are presented in supplementary information (Supplementary Figure S3).

IGTP overexpression can activate the PI3K/Akt signaling pathway in HeLa cells^29^. To examine the effect of PI3K/Akt on bacterial invasion into TG cells, TG cells were treated with wortmannin, a PI3K inhibitor, and with Akt 1/2 kinase inhibitor. Bacterial invasion was reduced by treatment of TG cells with PI3K/Akt inhibitors, with or without IFN-γ treatment (Fig. 4a). In addition, IFN-γ treatment induced the phosphorylation of Akt (p-Akt) in TG cells. Phosphorylation of Akt was reduced by treatment of PI3K/Akt inhibitors in TG cells with or without IFN-γ treatment (Fig. 4b). To examine the effect of IGTP on the activation of Akt, we used siRNA to knockdown IGTP in TG cells. The amount of p-Akt was reduced by the knockdown of IGTP (Fig. 5), suggesting that IGTP contributes to Akt activation.

### Lipid rafts contribute to bacterial internalization into TG cells

**Figure 6:**
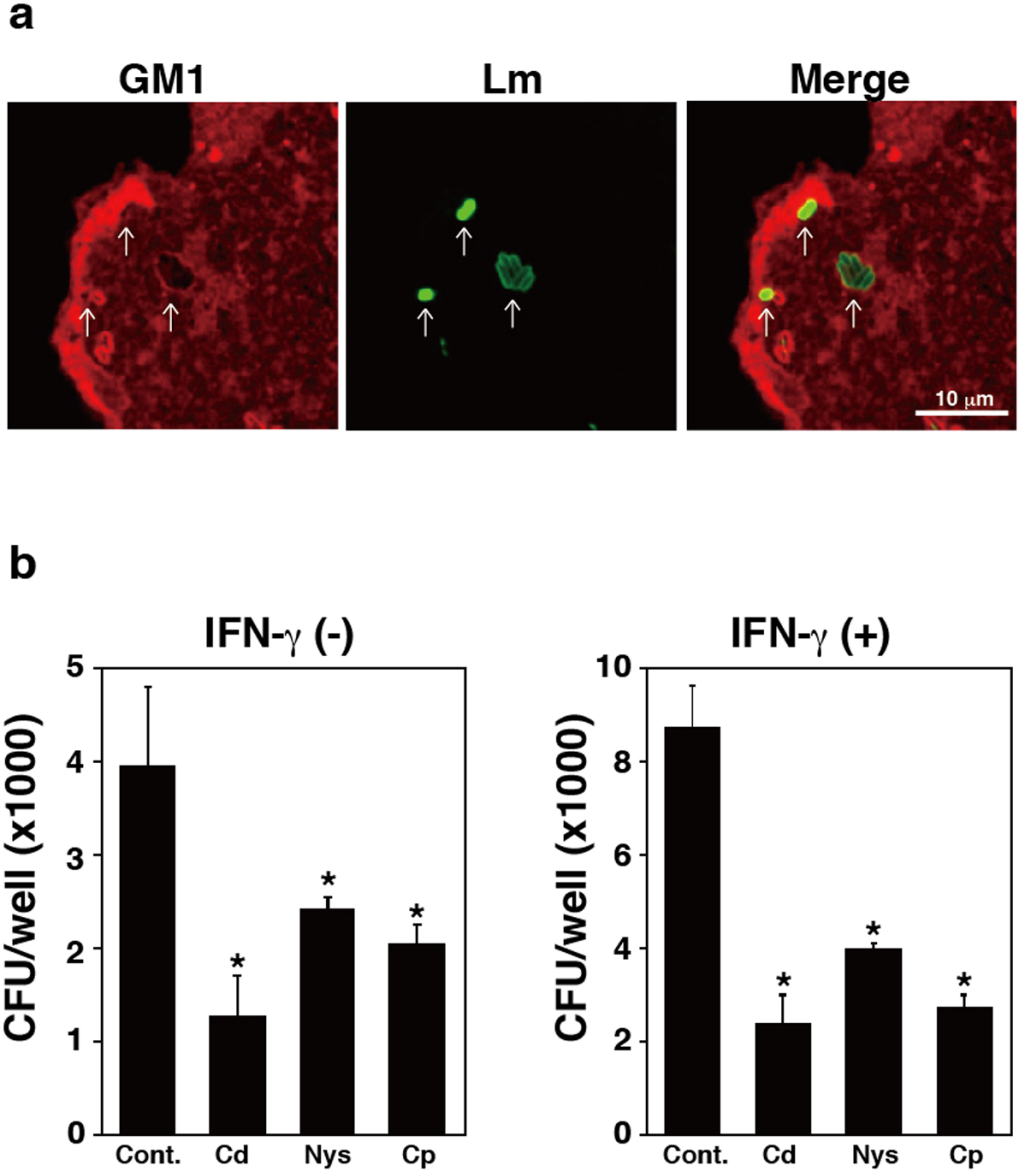
Localization of *L. monocytogenes* and GM1 ganglioside in TG cells using immunofluorescence microscopy. (a) TG cells were infected with *L. monocytogenes* for 30 min, then fixed and stained for GM1 ganglioside (red) and intracellular bacteria (green). Arrows indicate colocalized bacteria with GM1 ganglioside. Scale bar indicates 10 μm. (b) TG cells were treated with β-cyclodextrin (Cd, 5 μg/ml), Nystatin (Nys, 20 μg/ml), or chlorpromazine (Cp, 15 μg/ml) for 1 h. Treated TG cells were infected with *L. monocytogenes*in the presence or absence of IFN-γ (3,000 units/ml) for 24 h. Bacterial invasion into TG cells was measured using a bacterial invasion assay. Data represent the averages and standard deviations of triplicate samples from three identical experiments. Statistically significant differences between control and treated groups are indicated by asterisks (*, *P* < 0.05).

**Figure 7:**
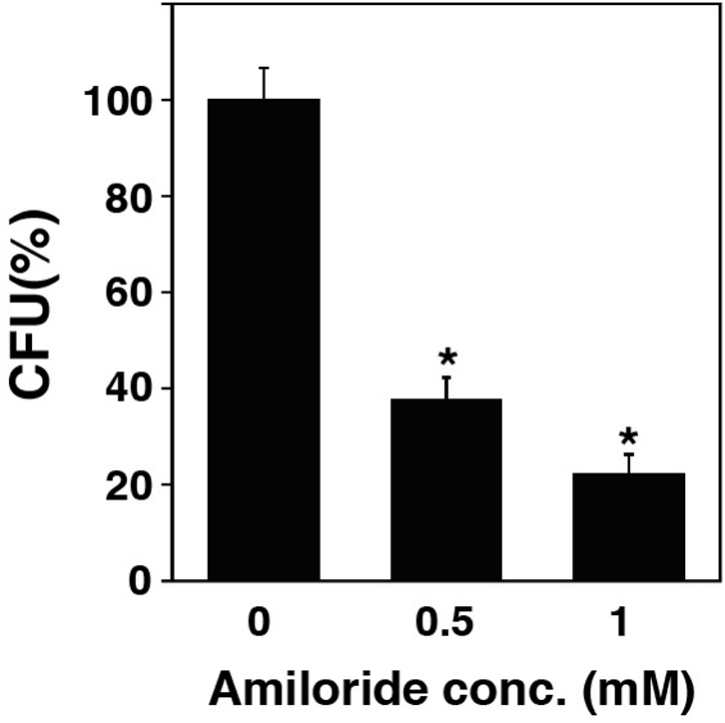
Macropinocytosis is associated with *L. monocytogenes* invasion into TG cells. TG cells were treated with amiloride at the indicated concentrations for 1 h. Treated TG cells were then infected with *L. monocytogenes*. Bacterial invasion into TG cells was measured using a bacterial invasion assay. Data represent the averages and standard deviations of triplicate samples from three identical experiments. Statistically significant differences between control and treated groups are indicated by asterisks (*, *P* < 0.05).

Because lipid rafts mediate PI3K/Akt signaling^30^, we examined the localization of GM1 gangliosides, which are associated with lipid rafts, in *L. monocytogenes*-infected TG cells. GM1 gangliosides accumulated in the membrane of *L. monocytogenes*-containing vacuoles in TG cells (Fig. 6a). Furthermore, bacterial invasion was reduced by treatment of TG cells with lipid raft inhibitors, such as β-cyclodextrin, nystatin, and chlorpromazine, with or without IFN-γ treatment (Fig. 6b). Because several intracellular pathogens infect into host cells using lipid raft-mediated macropinocytosis^31^,^32^, we assessed the effect of the macropinocytosis inhibitor, amiloride, on the invasion of *L. monocytogenes* into TG cells. Amiloride also inhibited bacterial invasion into TG cells (Fig. 7). These results suggested that lipid raft-mediated macropinocytosis contributes to the invasion of *L. monocytogenes* into TG cells.

## Discussion

It is well known that *L. monocytogenes* infection induces IFN-γ production and that IFN-γ is the most important cytokine for host defense against *L. monocytogenes* because administration of recombinant IFN-γ enhances antilisterial resistance^33^,^34^ and administration of an anti-IFN-γ antibody eliminates this resistance^35^. Mice lacking IFN-γ or the IFN-γ receptor are highly susceptible to *L. monocytogenes* infection^36^,^37^. On the other hand, IFN-γ is known to be harmful for pregnancy because recent studies have shown that factors induced by bacterial infection, such as IFN-γ and “regulated on activation, normal T cell expressed and secreted” (RANTES), induce infectious abortion in *Brucella* infection^38^. However, the detailed mechanisms of infectious abortion remain unclear. It has also been reported that TG cells have a phagocytic activity and that this activity is enhanced by IFN-γ treatment^27^,^28^. Therefore, we thought that IFN-γ-inducible factors may be involved in the invasion of *L. monocytogenes* into TG cells.

IFN-γ induces resistance to bacterial infection through broad transcriptional programs involving a variety of genes, many of which remain uncharacterized^39^,^40^. IGTP is a member of the immunity-related GTPase family M (IRGM) subfamily of the family of IFN-γ-responsive GTPases (IRG). The IRG play a critical role in IFN-γ-induced resistance to intracellular pathogens. LRG-47 knockout mice are susceptible to a range of organism, including intracellular protozoa (*L. major*, *Trypanosoma cruzi*, and *T. gondii)* and intracellular bacteria (*L. monocytogenes*, *Salmonella typhimurium*, *Mycobacterium avium*, *M. tuberculosis*, and *Chlamydia trachomatis*)^39^,^41^. In contrast, IGTP knockout mice are susceptible to infection with *T. gondii*, *L. major*, and *C. trachomatis* but are resistant to *T. cruzi*, *L. monocytogenes*, *S. typhimurium*, *M. avium*, *M. tuberculosis*, murine cytomegalovirus, and Ebola virus^39^,^41^. In this study, we observed that the efficienciy of *L. monocytogenes* invasion was significantly reduced by siRNA mediated knockdown of IGTP in TG cells. We do not know if there is a direct relationship between the inhibition of bacterial invasion caused by knockdown of IGTP in TG cells and the resistance to bacterial infection exhibited by IGTP knockout mice. IGTP may contribute to the invasion of *L. monocytogenes* into other types of cells, such as macrophages and epithelial cells, and that may be involved in resistance to bacterial infection.

IGTP localizes predominantly to the endoplasmic reticulum, and has been found to be essential for host resistance to acute infection by *T. gondii*^42^. Elimination of *T. gondii* is dependent on IGTP and requires PI3K activity in macriphages^43^. IGTP can be seen clustered adjacent to *T. gondii*^44^. We also observed IGTP accumulated around infected *L. monocytogenes* in TG cells. Therefore, we suspected that bacterial invasion via IGTP may be mediated by the PI3K/Akt pathway. Indeed, we demonstrated that invasion of *L. monocytogenes* requires PI3K/Akt activity, suggesting that there are mediator molecules upstream of the PI3K/Akt pathway on the surface of TG cells. In our previous studies, several receptors expressed on TG cells contributed to the invasion of *L. monocytogenes*, such as Toll-like receptor (TLR) 2, class B scavenger receptor type 1 (SR-B1), and Mannose receptor, C type 1 (MRC1)^27^,^28^. Because the expression of these receptors was not induced by IFN-γ^27^,^28^, IFN-γ-mediated enhancement of bacterial invasion into TG cells may result from contributions by other cell surface receptors. On the other hand, TLR2 and SR-B1 are known to activate of PI3K^45^. Several receptors, including TLR2, SR-B1, and MRC1, may mediate the signals of bacterial invasion and activate the PI3K/Akt pathway in TG cells.

Signal transduction through cell surface receptors, including TLR2 and SR-B1, is involved in lipid rafts^46^,^47^. Lipid rafts are specialized areas of the plasma membrane which are enriched in cholesterol, sphingolipids, glycolipids, and glycosylphosphatidylinositol-linked proteins^48^,^49^. Several intracellular pathogens invade into host cells through lipid rafts^50^,^51^,^52^. Invasion of *L. monocytogenes* into epithelial cells also involves lipid rafts^53^. We observed that lipid rafts and macropinocytosis contributed to bacterial invasion into TG cells in this study. These results suggested that putative cell surface receptors in lipid rafts mediate the signaling necessary for bacterial invasion into TG cells and activate the PI3K/Akt pathway, and that this signaling is enhanced by IFN-γ.

The PI3K/Akt pathway is also known to be involved in the expression of heme oxygenease-1 (HO-1)^54^. HO-1 plays key roles in cytoprotection, anti-oxidation, and anti-inflammation^55^,^56^. HO-1 expression in TG cells is decreased by *L. monocytogenes* infection and by treatment with IFN-γ. The reduction of HO-1 expression induces apoptosis in TG cells^9^. TG cell death inhibits the maintenance of pregnancy and results in infectious abortion. The modulation of the production of IFN-γ and IRG family members is an important host response for fetal survival and pathogen control. However, *L. monocytogenes* exploits this host defense system to achieve the survival strategy of the bacterium in the pregnant host. Recently, it has been reported that *L. monocytogenes* EGD strain has a point mutation in transcriptional regulator PrfA (PrfA*), leading to constitutive expression of several major virulence genes^57^. Since the mutation may affect invasion of *L. monocytogenes* into TG cells *in vitro* and *in vivo*, further analysis of bacterial virulence factors is also needed to clarify mechanism of bacterial invasion into TG cells.

## Methods

### Bacterial strains

*L. monocytogenes* EGD was maintained as a frozen glycerol stock and cultured in brain heart infusion (BHI) broth (Becton Dickinson, Franklin Lakes, NJ) or on BHI broth containing 1.5% agar.

### Cell culture

The mouse TS cell line was a gift from Dr. Tanaka^58^,^59^. TS cells were cultured in a mixed medium comprising a 3:7 ratio of TS medium to mouse embryonic fibroblast-conditioned medium, supplemented with 25 ng/ml fibroblast growth factor 4 (TOYOBO, Osaka, Japan) and 1 μg/ml heparin (Sigma, St. Louis, MO), as described in our previous study^59^. TS medium was prepared by adding 20% fetal bovine serum (FBS), 1 mM sodium pyruvate, 100 μM β-mercaptoethanol, and 2 mM l-glutamine to RPMI 1640 medium. To induce differentiation of TS cells to TG cells, TS cells were cultured in the TS medium alone for 3 days at 37°C in an atmosphere containing 5% CO_2_. TG cells were seeded (1–2 × 10^5^ per well) in 48-well tissue culture plates. Size of TG cells is clearly larger than TS cells and the differentiation was usually confirmed by microscopy.

### Efficiency of bacterial invasion into cultured cells

Overnight culture of *L. monocytogenes* strains were deposited onto TG cells at a multiplicity of infection (MOI) of 10 by centrifugation at 150 × g for 10 min at room temperature. To measure the efficiency of bacterial uptake, the infected cells were incubated at 37°C for 30 min, washed once with TS medium, and then incubated in TS medium containing gentamicin (50 µg/ml) for 30 min. The cells were washed thrice with phosphate-buffered saline (PBS) and lysed with cold distilled water. Colony forming units (CFUs) were determined by serial dilution on BHI agar plates. Wortmannin (5 μM, Sigma), Akt 1/2 kinase inhibitor (20 μM, Sigma), nystatin (20 μg/ml, Wako, Osaka, Japan), β-cyclodextrin (5 μg/ml, Wako), chlorpromazine (15 μg/ml, Wako), or amiloride (indicated concentrations, Sigma) was added to the RPMI 1640 medium 1 h before infection. Recombinant IFN-γ (3,000 units/ml, Cedarlane Laboratories, Ontario, Canada) was added to the TS medium 24 h before infection.

### Immunoblotting

TG cells were washed twice with PBS and lysed in lysis buffer [ice-cold Tris-buffered saline (TBS) containing 1% Triton X-100, 0.1% sodium dodecyl sulfate, 1% sodium deoxycholate, 10 mM EDTA, and 1× Halt Protease Inhibitor Cocktail Kit (Thermo Fisher Science, Rockford, IL)] at 4°C for 60 min. The cell lysates were centrifuged (13,200 rpm, 4°C, 30 min) and supernatants were collected. Protein concentrations were determined using the Bio-Rad Protein Assay (Bio-Rad, Richmond, CA). Protein samples were separated on 10% polyacrylamide gels and transferred to a polyvinylidene difluoride membranes, which were incubated for overnight at 4°C with anti-mouse IGTP goat antibody (1:1000, Santa Cruz Biotechnology, Dallas, TX), anti-mouse Akt rabbit antibody (1:2000, Cell Signaling, Beverly, MA), or anti-mouse phospho-Akt rabbit antibody (1:2000, Cell Signaling) in 1% skim milk. The membranes were then washed thrice in TBS with 0.02% Tween 20, incubated for 30 min with a horseradish peroxidase (HRP)-conjugated secondary antibody (0.01 μg/ml) at room temperature and washed again. Immunoreactions were visualized using the enhanced chemiluminescence detection system (GE Healthcare Life Science, Little Chalfont, UK). The anti-mouse β-actin antibody was purchased from Sigma. Immunoblots were quantified using the ImageJ 1.32 software (National Institutes of Health, Bethesda, MD) after densitometric scanning of the films by LAS-3000 Imaging System (Fujifilm Life Science, Tokyo, Japan).

### siRNA experiment

The siRNA duplexes used to silence mouse IGTP (target sequence:5′-TCCCATGGATTTAGTCACAAA-3′) and AllStars Negative Control siRNA which was used as a scrambled control were purchased from QIAGEN (Hilden, Germany). TG cells were transiently transfected using the Lipofectamine® RNAiMAX Transfection Reagent (Invitrogen, Carlsbad, CA) with or without siRNAs at a final concentration of 1 nM.

### RNA isolation and reverse transcription (RT)-PCR

Total RNA was isolated from TG cells using the RNAeasy Plus Mini Kit (QIAGEN). Purified RNA samples were stored at −80°C prior to use. The RNA was quantified by absorption at 260 nm using a SmartSpec3000 spectrophotometer (Bio-Rad). RT-PCR was carried out using the SuperScript One-Step RT-PCR kit with Platinum Taq polymerase (Invitrogen). The primer sequences were as follows. IGTP: 5′-GCTGCTCCTGCCTCTTCTAA-3′ and 5′-ATTTAGACCACGGGCTGATG-3′ (in this study) and β-actin: 5′-TGGAATCCTGTGGCATCCATGAAAC-3′ and 5′-TAAAACGCAGCTCAGTAACAGTCCG-3′^58^.

### Immunofluorescence microscopy

Bacteria were deposited onto TG cells grown on coverslips by centrifugation at 150 × g for 5 min at room temperature. The coverslips were then incubated at 37°C for 30 min. The samples were washed twice with PBS, fixed with 4% paraformaldehyde in PBS for 30 min at room temperature, washed thrice with PBS, and then successively incubated thrice for 5 min in blocking buffer (5% bovine serum albumin in PBS) at room temperature. The samples were permeabilized with 0.2% Triton X-100 and washed thrice with PBS, and then treated with 5 µg/ml anti-*L. monocytogenes* rabbit antibody (Viro Stat, Portland, ME) diluted in blocking buffer to identify intracellular bacteria or anti-mouse IGTP goat antibody to identify IGTP. After incubation for 1 h at 37°C, the samples were washed thrice for 5 min with blocking buffer, stained with TRITC-labeled goat anti-rabbit IgG (Santa Cruz Biotechnology) or FITC-labeled donkey anti-goat IgG (Santa Cruz Biotechnology) in blocking buffer, and incubated for 1 h at 37°C. Fluorescent images were obtained using a FluoView FV100 confocal laser scanning microscope (Olympus, Tokyo, Japan). To detect the localization of GM1 gangliosides, TG cell monolayers were incubated for 5 min with biotin-cholera toxin B subunit (CTB) (10 μg/ml, List Biological Laboratories, Campbell, CA), rinsed thrice in RPMI 1640 medium, and then incubated with *L. monocytogenes* for 30 min at 37°C. The cells were washed once, fixed in periodate-lysine-paraformaldehyde (PLP)-sucrose and probed for extracellular bacteria, as above, before permeablization in 0.2% Triton X-100 for 20 min at room temperature. After three washes with PBS and incubation in blocking buffer, the biotin-CTB was detected using Alexa Fluor 594-streptavidin (1:500, Molecular Probes, Eugene, OR)^32^.

### Statistical analysis

Statistical analyses were performed using Student's *t*-test. Statistically significant differences, compared with control, are indicated by asterisks (*, *P* < 0.05). Data are the averages of triplicate samples from three identical experiments and the error bars represent standard deviations.

## Author Contributions

M.T. and M.W. planned the experiments and wrote the main manuscript. M.H., K.W., and T.S. discussed the experimental findings and interpretation of the results. M.T., M.H., and K.W. participated in the immunological and genetic experiments. M.T., T.S., and M.W. participated in the cell biology experiments.

## Supplementary Material

Supplementary Information Supplementary information
