# Supplementary material for: Interferon γ-induced GTPase promotes invasion of Listeria monocytogenes into trophoblast giant cells
Source: Sci Rep. 2015 Feb 3;5:8195. doi: 10.1038/srep08195 (PMC4314643; doi:10.1038/srep08195)
Supplement: Supplementary Information [file srep08195-s1.doc]

Supplementary information for

Interferon γ-induced GTPase promotes internalization of *Listeria monocytogenes* into trophoblast giant cells

Masato Tachibana, Masanori Hashino, Kenta Watanabe, Takashi Shimizu, and Masahisa Watarai*

The United Graduate School of Veterinary Science, Yamaguchi University, Yamaguchi, Japan

*Correspondence author:

Dr. Masahisa Watarai,

The United Graduate School of Veterinary Science, Yamaguchi University,

1677-1 Yoshida, Yamaguchi 753-8515, Japan.

E-mail: watarai@yamaguchi-u.ac.jp

Supplementary Figure S1 is related to Figure 2.

Supplementary Figure S2 is related to Figure 4b.

Supplementary Figure S3 is related to Figure 5.


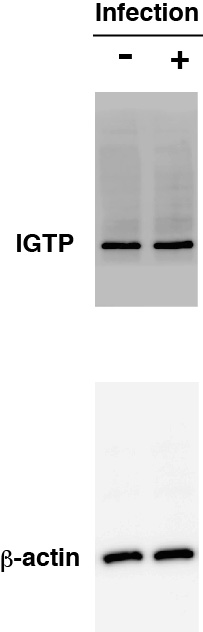


**Supplementary Figure S1** *L. monocytogenes* infection does not affect IGTP expression in TG cells. TG cells were infected with (+) or without *L. monocytogenes* (-); IGTP expression was analyzed by immunoblotting. β-actin was used as a control.


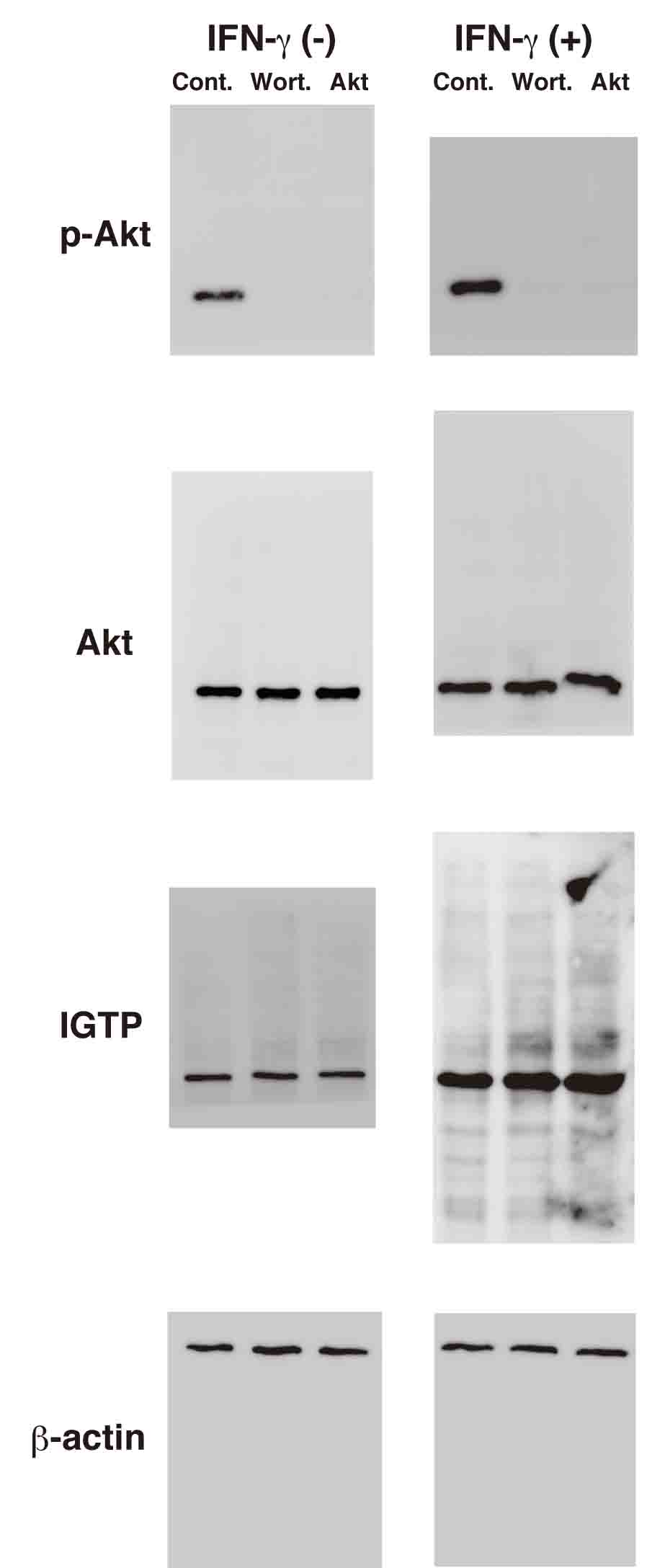


**Supplementary Figure S2** Full length images of immunoblotting in Figure 4b. TG cells were treated for 1 h with wortmannin or Akt 1/2 kinase inhibitor with or without IFN-γ, for 24 h. Expression of the indicated proteins was detected by immunoblotting. β-actin was used as a control.


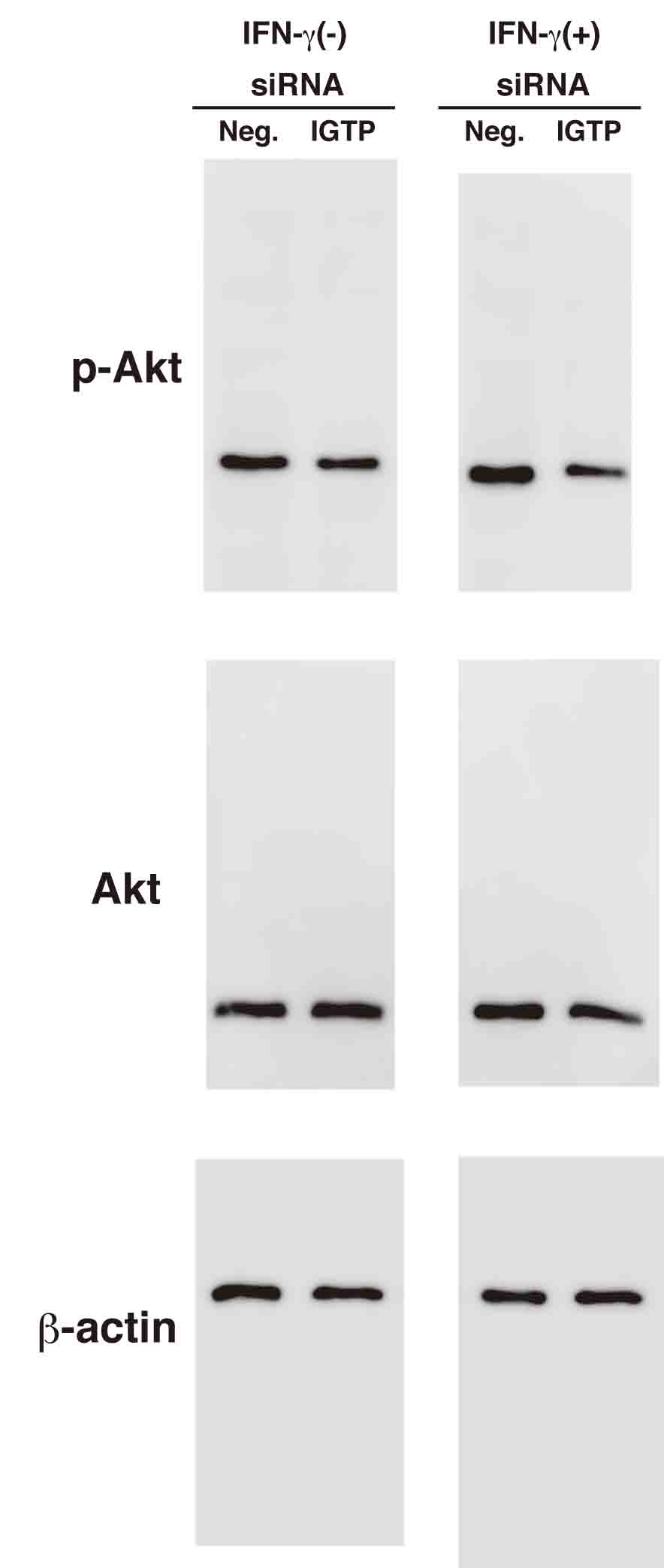


**Supplementary Figure S3** Full length images of immunoblotting in Figure 5. TG cells were treated for 48 h with either siRNA-targeting IGTP, with or without IFN-γ, for 24 h (IGTP). A negative control sample was treated with AllStars Negative Control siRNA (Neg.). Expression of the indicated proteins was detected by immunoblotting. β-actin was used as a control.
